# Oxygenation Threshold Derived from Near-Infrared Spectroscopy: Reliability and Its Relationship with the First Ventilatory Threshold

**DOI:** 10.1371/journal.pone.0162914

**Published:** 2016-09-15

**Authors:** Stephan van der Zwaard, Richard T. Jaspers, Ilse J. Blokland, Chantal Achterberg, Jurrian M. Visser, Anne R. den Uil, Mathijs J. Hofmijster, Koen Levels, Dionne A. Noordhof, Arnold de Haan, Jos J. de Koning, Willem J. van der Laarse, Cornelis J. de Ruiter

**Affiliations:** 1 Department of Human Movement Sciences, Vrije Universiteit Amsterdam, MOVE Research Institute Amsterdam, the Netherlands; 2 Department of Physiology, VU Medical Center, Amsterdam, the Netherlands; 3 Faculty of Sports and Nutrition, Amsterdam University of Applied Sciences, Amsterdam, the Netherlands; University of Alabama at Birmingham, UNITED STATES

## Abstract

**Background:**

Near-infrared spectroscopy (NIRS) measurements of oxygenation reflect O_2_ delivery and utilization in exercising muscle and may improve detection of a critical exercise threshold.

**Purpose:**

First, to detect an oxygenation breakpoint (Δ[O_2_HbMb-HHbMb]-BP) and compare this breakpoint to ventilatory thresholds during a maximal incremental test across sexes and training status. Second, to assess reproducibility of NIRS signals and exercise thresholds and investigate confounding effects of adipose tissue thickness on NIRS measurements.

**Methods:**

Forty subjects (10 trained male cyclists, 10 trained female cyclists, 11 endurance trained males and 9 recreationally trained males) performed maximal incremental cycling exercise to determine Δ[O_2_HbMb-HHbMb]-BP and ventilatory thresholds (VT1 and VT2). Muscle haemoglobin and myoglobin O_2_ oxygenation ([HHbMb], [O_2_HbMb], SmO_2_) was determined in m. vastus lateralis. Δ[O_2_HbMb-HHbMb]-BP was determined by double linear regression. Trained cyclists performed the maximal incremental test twice to assess reproducibility. Adipose tissue thickness (ATT) was determined by skinfold measurements.

**Results:**

Δ[O_2_HbMb-HHbMb]-BP was not different from VT1, but only moderately related (r = 0.58–0.63, p<0.001). VT1 was different across sexes and training status, whereas Δ[O_2_HbMb-HHbMb]-BP differed only across sexes. Reproducibility was high for SmO_2_ (ICC = 0.69–0.97), Δ[O_2_HbMb-HHbMb]-BP (ICC = 0.80–0.88) and ventilatory thresholds (ICC = 0.96–0.99). SmO_2_ at peak exercise and at occlusion were strongly related to adipose tissue thickness (r^2^ = 0.81, p<0.001; r^2^ = 0.79, p<0.001). Moreover, ATT was related to asymmetric changes in Δ[HHbMb] and Δ[O_2_HbMb] during incremental exercise (r = -0.64, p<0.001) and during occlusion (r = -0.50, p<0.05).

**Conclusion:**

Although the oxygenation threshold is reproducible and potentially a suitable exercise threshold, VT1 discriminates better across sexes and training status during maximal stepwise incremental exercise. Continuous-wave NIRS measurements are reproducible, but strongly affected by adipose tissue thickness.

## Introduction

Performance benefits from adequate training. Even though training intensity can be quantified in many different ways, endurance training intensity is often quantified by the lactate thresholds (LT1 and LT2) obtained from blood sampling or the ventilatory thresholds (VT1 and VT2) obtained from gas exchange data [1]. Above LT1 and VT1, blood lactate concentration is elevated [1] and CO_2_ production exceeds oxygen uptake due to anaerobic energy production in the muscle cells as bicarbonate buffers the produced lactic acid [2]. Above LT2 and VT2, blood lactate accumulates as lactate production exceeds removal rates [1] and respiratory compensation starts by an excessive increase in minute ventilation [3]. There is consensus that one can obtain ventilatory or lactate thresholds reproducibly from an incremental exercise test [1,4,5] and can use these thresholds for training purposes [1]. However, ventilatory and lactate thresholds are derived from measurements at the mouth or (closer to the muscle) in the blood. Therefore, the first ventilatory and lactate threshold rather indirectly and with a delay reflect an increase of anaerobic ATP resynthesis. Both exercise intensity and oxygen availability contribute to this transition. Increases in the former lead to recruitment of additional (larger) motor units with lower oxidative capacity, while the latter depends on O_2_ consumption by the contracting muscle fibres and O_2_ supply by the O_2_ cascade from the air to the mitochondria [6]. Near-infrared spectroscopy (NIRS) provides non-invasive measures of the balance between oxygen delivery and consumption in the muscle [7], and thereby NIRS is potentially a very suitable technique to detect a critical exercise threshold directly in the exercising muscle.

In active muscle, the matching of oxygen supply ($\dot{Q}O_{2}$) and oxygen consumption ($m\dot{V}O_{2}$) is crucial for *O*_2_ diffusion and metabolic control [8]. This $\dot{Q}O_{2}/m\dot{V}O_{2}$ matching can theoretically be assessed non-invasively by NIRS [8]. Hereto, concentration changes of deoxygenated haemoglobin and myoglobin ([HHbMb]), oxygenated haemoglobin and myoglobin ([O_2_HbMb]) and tissue saturation (SmO_2_) are measured. These NIRS signals present multiple breakpoints during incremental exercise. For instance, the [HHbMb] breakpoint during ramp exercise is suggested to resemble an upper limit of fractional oxygen extraction in the exercising leg muscles during incremental exercise [9–11] and approximates VT2 [10,12,11,13–15], maximal lactate steady state [14,16] and critical power [14]. At somewhat lower exercise intensities, a breakpoint in Hb difference ([O_2_HbMb-HHbMb]) occurs during maximal stepwise incremental exercise and shows high correlations with VT1 and the lactate threshold (r>0.88) [17–19]. At this [O_2_HbMb-HHbMb] breakpoint, the fractional oxygen extraction supposedly has not yet reached its upper limit in the exercising leg muscles during incremental exercise. Therefore, this breakpoint could reflect a transition in oxygen extraction that is likely attributed to capillary-venular PO_2_ reaching its critical value (PO_2crit_) and lactic acidosis inducing a rightward shift of the O_2_Hb dissociation curve (i.e. Bohr effect) [17]. Hence, the [O_2_HbMb-HHbMb] breakpoint may relate to the occurrence of oxygen limitation in the skeletal muscle fibres.

Although reproducibility of ventilatory thresholds [4] and lactate threshold [5] are known to be high, data on reproducibility of NIRS signals [5,20–22] and NIRS derived breakpoints [23] is limited. Only in a single study with sedentary subjects, reproducibility of breakpoints in [O_2_HbMb] has been determined (r = 0.67–0.85) [23]. However, it is yet unknown whether reproducibility of these [O_2_HbMb] breakpoints also applies to trained subjects and to other NIRS derived breakpoints.

At present, the [O_2_HbMb-HHbMb] breakpoint has been determined during cycling exercise in mountain climbers [17], fin swimmers [18] and college students [19]. However, the [O_2_HbMb-HHbMb] breakpoint has not been assessed with subjects differing in training status and sex in one study. It is important to note that adipose tissue thickness (ATT) may vary widely between male and female subjects and across subjects with heterogeneous training status. Also, it is well-known that scattering and absorbance due to adipose tissue affect NIRS signals, reducing absorbance by underlying muscle tissue [24–26]. One may reduce these effects of ATT by normalizing oxygenation changes to the oxygenation at peak exercise [10,27], maximal voluntary contraction [26,28] or cuff occlusion [21,26], where the effect of ATT on the amplitude of separate [O_2_HbMb] and [HHbMb] signals can be assessed in the absence of blood volume changes.

The main purpose of this study was to investigate how the Δ[O_2_HbMb-HHbMb] breakpoint relates to ventilatory thresholds during incremental step exercise across sexes and training status. Secondary purposes of the study were to determine the reproducibility of NIRS signals and exercise thresholds and to assess the confounding effect of adipose tissue thickness on NIRS signals. We expected that exercise intensity is similar at the [O_2_HbMb-HHbMb] breakpoint and VT1. In addition, we hypothesized that both the [O_2_HbMb-HHbMb] breakpoint and VT1 differ across sexes and training status due to differences in oxygen supply [29–32] and oxygen utilization [29,30] within the exercising muscles.

## Methods

### Subjects

Forty subjects participated in the present study: 10 trained male cyclists (CM), 10 trained female cyclists (CF), 11 endurance trained males (EM) and 9 recreationally trained males (RM) as classified by their $\dot{V}O_{2max}$ [33] (characteristics are summarized in Table 1). Female and male cyclists (CF, CM) were recruited from Dutch cycling teams, and RM and EM were recruited based on training volume (EM >5 hours endurance training per week and RM <3 hours training per week). Note that EM contained 7 cyclists and 4 non-cyclists, since many endurance athletes perform cycling exercise in the Netherlands. Reliability measurements were performed in CM and CF. Prior to participation, experimental procedures, risks and aims of the study were explained and all subjects provided written informed consent. The study was conducted according to the principles of the Declaration of Helsinki and was approved by the ethics committee of the Department of Human Movement Sciences, Vrije Universiteit, Amsterdam, the Netherlands.

**Table 1:** Subject characteristics.

Characteristic	CM (n = 10)	CF (n = 10)	EM (n = 11)	RM (n = 9)
Age (y)	23±3	24±4	23±2	24±2
Weight (kg)	79.2±5.2 ^b^	63.6±4.2 ^a^^,^^c^^,^^d^	80.5±7.1 ^b^	81.2±10.3 ^b^
ATT VL (mm)	3.3±0.8 ^b^	8.4±2.1 ^a^^,^^c^^,^^d^	3.5±1.2 ^b^	5.6±3.1 ^b^
Cycling experience (y)	4.8±2.6	6.3±3.6	N/A	N/A
Training volume (h•wk^-1^)	11.0±3.5 ^d^	9.6±4.0 ^d^	8.8±3.8 ^d^	2.6±1.4 ^a^^,^^b^^,^^c^
No. of training sessions (wk^-1^)	3.7±1.5 ^d^	4.3±1.2 ^d^	4.7±2.3 ^d^	1.9±1.2 ^a^^,^^b^^,^^c^
$\dot{V}O_{2max}$ (ml•kg^-1^ •min^-1^)	60.0±6.6 ^d^	53.6±4.5 ^c^	60.8±5.5 ^b^^,^^d^	48.7±5.3 ^a^^,^^c^
$\dot{V}O_{2max}$ (L•min^-1^)	4.75±0.56 ^b^^,^^d^	3.39±0.22 ^a^^,^^c^^,^^d^	4.88±0.35 ^b^^,^^d^	3.91±0.27 ^a^^,^^b^^,^^c^
PO_peak_ (W)	356±41 ^b^^,^^d^	252±21 ^a^^,^^c^	359±36 ^b^^,^^d^	286±35 ^a^^,^^c^

a Indicates significantly different from male cyclists (p < 0.05).

b Indicates significantly different from female cyclists (p < 0.05).

c Indicates significantly different from endurance trained males (p < 0.05).

d Indicates significantly different from recreationally trained males (p < 0.05).

### Experimental design

Subjects performed a maximal incremental step exercise test to exhaustion on a friction-braked cycle ergometer (Monark Ergomedic 839E, Monark exercise AB, Sweden), pedalling at 90 rpm. After three minutes of seated rest, subjects started at a workload of 1.5W·kg^-1^ (85–145W), which was increased with 0.5W·kg^-1^ every three minutes (30–50W·3min^-1^) until voluntary exhaustion. Trained cyclists performed two maximal incremental tests on separate days to assess reproducibility of NIRS signals and exercise thresholds. The test was terminated if cadence dropped below 80 rpm, despite verbal encouragement. Active cool-down consisted of five minutes cycling at 1 W·kg^-1^ (60–100W). Thereafter, an arterial occlusion was performed to assess maximal desaturation of the m. vastus lateralis. Hereto, a pressure cuff was inflated to approximately ~300mmHg at the most proximal part of the thigh until maximal desaturation was reached (duration 314 ± 44 s). The cuff was secured by an extra strap that prevented it from unwrapping [34].

Prior to the maximal incremental test, subjects were instructed to avoid strenuous exercise and alcohol consumption within 24 hours before the test and to consume their last meal and caffeinated beverages at least three hours prior to testing. Subsequent measurements were separated by at least one day of rest. Saddle and handle bar position were adjusted to individual preferences and were recorded for subsequent measurements. Environmental circumstances were controlled in a climate-controlled environment (temperature 16.3±0.9°C, relative humidity 46.4±6.4%).

### Data collection

#### Pulmonary measures

Ventilation and gas exchange were analysed breath-by-breath using open circuit spirometry (Cosmed Quark CPET, Cosmed S. R. L., Rome, Italy). Prior to every test, the gas analyser and volume transducer were calibrated according to the manufacturer’s instructions.

#### Near-Infrared Spectroscopy

Relative changes with respect to the start of maximal incremental test were measured for haemoglobin + myoglobin ([HHbMb], [O_2_HbMb]) and muscle saturation (SmO_2_) by a continuous-wavelength portable NIRS device using spatially resolved spectroscopy (Portamon, Artinis Medical Systems, Arnhem, the Netherlands). Concentration changes of the right leg were determined at 10Hz at the vastus lateralis (VL) muscle belly and calculated from light absorbance at 758nm and 847nm using the modified Lambert-Beer law. The NIRS device was positioned above the upper patella border at 1/3 distance of the patella and greater trochanter, parallel to the longitudinal femur axis. Light emitting optodes were positioned at 30, 35 and 40mm distal to the receiver, sending near-infrared light into the thigh with a corresponding penetration depth of 15–20mm [24]. How much light the muscle tissue can absorb depends on light absorbance and scattering by skin and subcutaneous adipose tissue (e.g. influenced by adipose tissue thickness, temperature, and pigment) and on secured contact of the NIRS device with the skin. Motion artefacts were minimized by fixating the optode position using adhesive tape (Fixomull®) and elastic bandages. Additionally, placement of a light absorbing cloth over the NIRS device minimized the influence from ambient light. Finally, probe position was marked for replication in subsequent measurements.

#### Skinfold

Skinfold thickness of the m. vastus lateralis at the site of the NIRS device was measured (median of three measurements) in seated position using Harpenden skinfold callipers (British Indicators Ltd, Burgess Hill, UK). Adipose tissue thickness was calculated by dividing skinfold thickness by two [25], resembling subcutaneous fat and skin. Subjects showed a wide range in adipose tissue thickness (2.0–11.7 mm), yet participants were excluded if adipose tissue thickness was above 12.5 mm.

### Data analysis

Gas exchange, power output (PO) and NIRS data were converted to 1Hz samples using linear interpolation, filtered for extreme values (data points deviating > 2 SD from a local window of 6 data points were replaced by the local average) and time-assembled. Critical exercise thresholds and maximal physiological parameters were derived from 1Hz data using smoothed 30-second moving averages and peak PO was defined as average PO over the last 180 seconds prior to termination of the test. VT1 was derived from respiratory exchange measurements using the V-slope method and ventilatory equivalents [35] and VT2 was derived using the minute ventilation versus $\dot{V}{CO}_{2}$ graph and ventilatory equivalents [3]. Both thresholds were detected by automatic regression and subsequently (re)evaluated by two independent observers. In addition, the difference between concentration changes in [HHbMb] and [O_2_HbMb] were used to derive an oxygenation breakpoint (Δ[O_2_HbMb-HHbMb]-BP) using a double linear regression method [17–19]. Both linear regressions were fitted through Δ[O_2_HbMb-HHbMb] versus relative PO (%PO) plots. Δ[O_2_HbMb-HHbMb]-BP was defined as the intercept of two congregating regression lines with the least combined residuals sum of squares (Eq 1). Warm-up stages were excluded and regression lengths were set at ≥10% of relative workload.

$$\begin{array}{l} y_{1}=m_{1}∙x+b_{1}\;for\;x<BP\\ y_{2}=m_{2}∙(x-BP)+(m_{1}∙BP+b_{1})\;for\;x>BP\\ y_{1}(BP)=y_{2}(BP)\\ BP=\Delta {[O}_{2}HbMb-HHbMb]-BP \end{array}$$ (1)

In addition, the confounding effect of ATT on NIRS signals was determined for SmO_2_ at the end of maximal exercise and arterial occlusion. The amplitudes of [HHbMb] and [O_2_HbMb] were assessed during rest and at the end of maximal exercise and arterial occlusion to determine the symmetry of these NIRS signals in relation to ATT. Moreover, next to the SmO_2_ values provided by the NIRS apparatus, SmO_2_ was also normalized to maximal changes during exercise (Eq 2). All data processing procedures were performed using custom written software in Matlab (Mathworks Co, Natick, Massachusetts, USA).

$${normalized\;SmO}_{2}=\frac{{SmO}_{2}-{SmO}_{2,rest}\;}{{SmO}_{2,rest}-{SmO}_{2,max}}$$ (2)

### Statistics

All data are presented as individual values or as mean±SD, unless indicated otherwise. Differences in $\dot{V}O_{2}$ and PO between Δ[O_2_HbMb-HHbMb]-BP and ventilatory thresholds were assessed by paired samples t-test and Pearson’s product-moment correlation analyses. A priori sample size calculations have been performed in GPower 3.1.2 (University Kiel, Germany) using the t-tests means (difference between two dependent means, matched pairs, a priori) with significance level 0.05 and power 80%. These calculations showed that differences in the Δ[O_2_HbMb-HHbMb]-BP and VT1 could be detected with a sample size of 9 subjects for PO (W) and 4–8 subjects for $\dot{V}O_{2}$ (L/min) [18,19]. Differences between groups were assessed by one-way ANOVAs (between factor group: CM-CF-EM-RM). Moreover, reproducibility was determined by intra class-correlations (i.e. single measures ICC_3,1_) and within-subject coefficient of variation (CV) after logarithmic transformation of the data. Qualification of correlation coefficients was performed according to Evans [36]. Relationships between ATT and SmO_2_ were assessed by hyperbolic regressions and relationships between ATT and the symmetry of changes in [HHbMb] and [O_2_HbMb] amplitude were assessed by linear regressions. Presented R-squared values were adjusted for the number of predictors (i.e. coefficients) in the regression model, being a more conservative measure of explained variance. Differences were considered to be significant if p < 0.05.

## Results

### Critical exercise threshold

**Fig 1:**
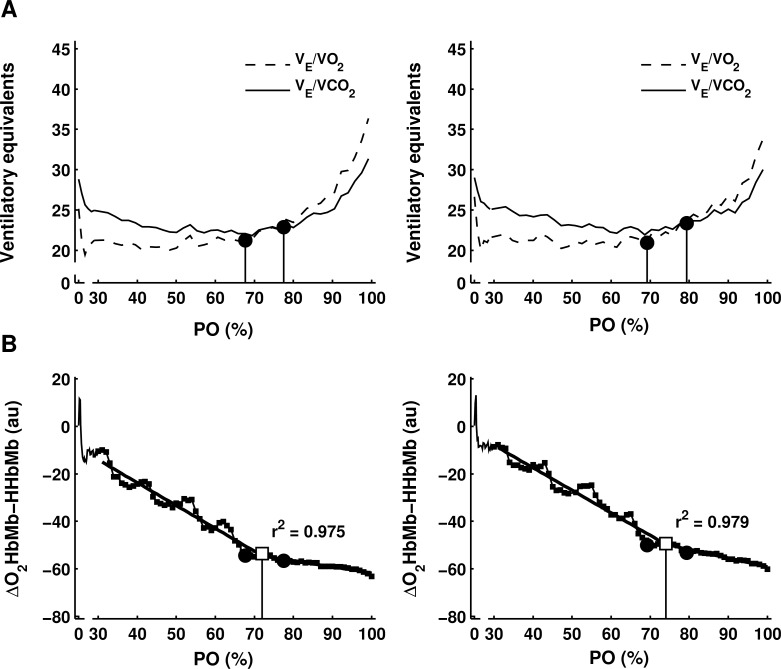
Typical example of detection of Δ[O_2_HbMb-HHbMb]-BP and ventilatory thresholds. Ventilatory thresholds VT1 and VT2 (closed circles) are depicted within the ventilatory equivalent versus PO graph (upper graphs, a). Δ[O_2_HbMb-HHbMb]-BP (open square) is derived from Δ([O_2_HbMb]–[HHbMb]) versus PO graph by means of double linear regression analyses with the least combined residual sum of squares (lower graphs, b). Both Δ[O_2_HbMb-HHbMb]-BP and ventilatory thresholds are determined for subsequent trials in a trained male cyclist for test (left graphs) and retest (right graphs).

**Fig 2:**
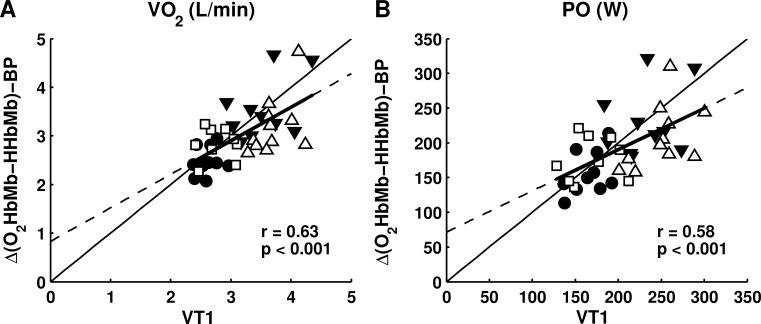
Relationship between Δ[O_2_HbMb-HHbMb]-BP and VT1. Δ[O_2_HbMb-HHbMb]-BP and VT1 are moderately related for $\dot{V}O_{2}$ (a) en PO (b). Individual values are shown for male cyclists (closed triangle), female cyclists (closed circle), endurance trained males (open square), recreationally trained males (open triangle). The thick solid line represents the best linear fit obtained from individual data at Δ[O_2_HbMb-HHbMb]-BP and VT1 and the dotted line represents extrapolation of this best linear fit. The thin solid line is the line of identity.

Δ[O_2_HbMb-HHbMb]-BP was determined with high regression model quality (r^2^ = 0.97 ± 0.02, Fig 1). Including all subjects, Δ[O_2_HbMb-HHbMb]-BP was not significantly different from VT1 ($\dot{V}O_{2}$: -0.155±0.509 L/min; PO: -12.1±44.2 W), but significantly lower than VT2 ($\dot{V}O_{2}$: -0.624±0.521 L/min; PO: -56.8±44.2W, p<0.001, Table 2). Subgroup analyses revealed that Δ[O_2_HbMb-HHbMb]-BP was not significantly different from VT1 in CM, CF and RM, however, in EM the Δ[O_2_HbMb-HHbMb]-BP was significantly smaller than VT1 (p<0.05). Δ[O_2_HbMb-HHbMb]-BP was only moderately related to VT1 ($\dot{V}O_{2}$: r = 0.63; PO: r = 0.58; p<0.001; Fig 2) and VT2 ($\dot{V}O_{2}$: r = 0.68; PO: r = 0.62; p<0.001). For subgroups, Δ[O_2_HbMb-HHbMb]-BP showed a better relationship with ventilatory thresholds in trained cyclists (r = 0.68–0.84, p<0.05) compared to endurance and recreationally trained males (r = 0.48–0.50, p<0.05). Although relative PO was not different between groups at Δ[O_2_HbMb-HHbMb]-BP, relative PO at VT1 was significantly lower in recreational males compared to other groups (RM< CM~EM~CF, p<0.05). Therefore, ventilatory thresholds differed across sexes and training status, whereas Δ[O_2_Hb-HHb]-BP differed only across sexes.

**Table 2:** Group differences in SmO_2_ values at rest, exercise and end arterial occlusion and in PO and $\dot{V}O_{2}$ values at the exercise thresholds.

	CM	CF	EM	RM
Rest SmO_2_ (%)	57±3.2 ^b^^,^^d^	63±4.0 ^a^	61±5.1	66±4.4 ^a^
Δ[O_2_HbMb-HHbMb]-BP SmO_2_ (%)	32±8.3 ^b^^,^^d^	56±5.8 ^a^^,^^c^	39±13.8 ^b^	46±13.2 ^a^
Δ[O_2_HbMb-HHbMb]-BP (W)	230±50 ^b^^,^^d^	156±31 ^a^^,^^c^	208±46 ^b^	177±32 ^a^
Δ[O_2_HbMb-HHbMb]-BP (%PO_peak_)	65±11	62±12	58±12	62±10
Δ[O_2_HbMb-HHbMb]-BP (L/min)	3.53±0.62 ^b^^,^^d^	2.49±0.29 ^a^^,^^c^	3.19±0.60 ^b^	2.83±0.33 ^a^
VT1 SmO_2_ (%)	34±8.3 ^b^^,^^d^	55±4.0 ^a^^,^^c^	35±14.1 ^b^^,^^d^	49±10.8 ^a^^,^^c^
VT1 (W)	232±34 ^b^^,^^d^	165±20 ^a^^,^^c^	250±30 ^b^^,^^d^	169±28 ^a^^,^^c^
VT1 (%PO_peak_)	65±5 ^d^	65±4 ^d^	69±3^d^	59±4 ^a^^,^^b^^,^^c^
VT1 (L/min)	3.52±0.45 ^b^^,^^d^	2.61±0.19 ^a^^,^^c^	3.70±0.31 ^b^^,^^d^	2.77±0.27 ^a^^,^^c^
VT2 SmO_2_ (%)	30±9.1 ^b^^,^^d^	54±4.1 ^a^^,^^c^	32±13.7 ^b^^,^^d^	45±12.1 ^a^^,^^c^
VT2 (W)	277±41 ^b^^,^^d^	199±18 ^a^^,^^c^	300±35 ^b^^,^^d^	218±33 ^a^^,^^c^
VT2 (%PO_peak_)	78±5 ^c^	79±4	84±3 ^a^^,^^d^	76±4 ^c^
VT2 (L/min)	4.03±0.58 ^b^^,^^d^	2.95±0.19 ^a^^,^^c^	4.27±0.37 ^b^^,^^d^	3.21±0.31 ^a^^,^^c^
Maxtest SmO_2_ (%)	26±11.5 ^b^^,^^d^	52±3.8 ^a^^,^^c^	28±14.0^b^^,^^d^	44±12.0 ^a^^,^^c^
Occlusion SmO_2_ (%)	25±8.3 ^b^	48±4.8 ^a^^,^^c^^,^^d^	24±12.4^b^	34±13.6 ^b^

Abbreviations: VT1, first ventilatory threshold; VT2, respiratory compensation threshold; SmO2, muscle saturation; Δ[O2HbMb-HHbMb]-BP, oxygenation breakpoint. Note that occlusion data were successfully obtained in 36 out of 40 subjects.

a Indicates significantly different from male cyclists (p < 0.05).

b Indicates significantly different from female cyclists (p < 0.05).

c Indicates significantly different from endurance trained males (p < 0.05).

d Indicates significantly different from recreationally trained males (p < 0.05).

### Reproducibility

Reproducibility was assessed in twenty trained cyclists (CM and CF). During the two maximal incremental tests, these cyclists displayed comparable $\dot{V}O_{2max}$ values (mean difference: -0.012±0.179 L/min) and peak workload (mean difference: -1.3±8W). In rest, during exercise and during occlusion, SmO_2_ measurements showed high reproducibility (mean differences between 0.5–1.8%, ICC = 0.69–0.97; Table 3). Moreover, reproducibility of $\dot{V}O_{2}$ and PO values was high at Δ[O_2_HbMb-HHbMb]-BP (ICC = 0.80–0.88) and excellent at VT1 and VT2 (ICC = 0.96–0.99; Table 3).

**Table 3:** Reproducibility of SmO_2_ values at rest, exercise and arterial occlusion and reproducibility of PO and $\dot{V}O_{2}$ at the exercise thresholds among twenty trained cyclists.

	Exercise intensity		Test	Retest	Differences Retest-Test	CV	ICC_(3,1)_
SmO_2_ (%)	Rest		60.2±4.8	59.7±4.2	-0.5±3.7	4.4	0.69
	Maximal incrementalexercise	50% $\dot{V}O_{2max}$	54.7±7.9	52.9±7.9	-1.8±3.7	5.2	0.90
		75% $\dot{V}O_{2max}$	45.5±12.6	44.9±12.0	-0.6±4.3	9.1	0.92
		100% $\dot{V}O_{2max}$	40.3±14.9	39.4±15.0	-0.8±3.8	8.0	0.97
	Occlusion		38.1±13.9	37.5±14.1	-0.6±4.0	9.0	0.97
PO (W)*	Δ(O_2_HbMb–HHbMb)-BP		193±55	203±54	10.2±31.4	13.0	0.80
	VT1		198±44	194±44	-4.1±12.6	4.7	0.96
	VT2		238±50	237±50	-0.8±8.0	2.8	0.98
$\dot{V}O_{2}$ (L/min)	Δ(O_2_HbMb–HHbMb)-BP		3.01±0.71	3.09±0.70	-0.09±0.32	8.4	0.88
	VT1		3.06±0.57	3.03±0.62	-0.03±0.15	3.6	0.97
	VT2		3.49±0.70	3.51±0.72	0.02±0.11	2.3	0.99

Abbreviations: VT1, first ventilatory threshold; VT2, respiratory compensation threshold; SmO2, muscle saturation; Δ[O2HbMb-HHbMb]-BP, oxygenation breakpoint. Note that occlusion data were successfully obtained in 36 out of 40 subjects.

### Adipose tissue

**Fig 3:**
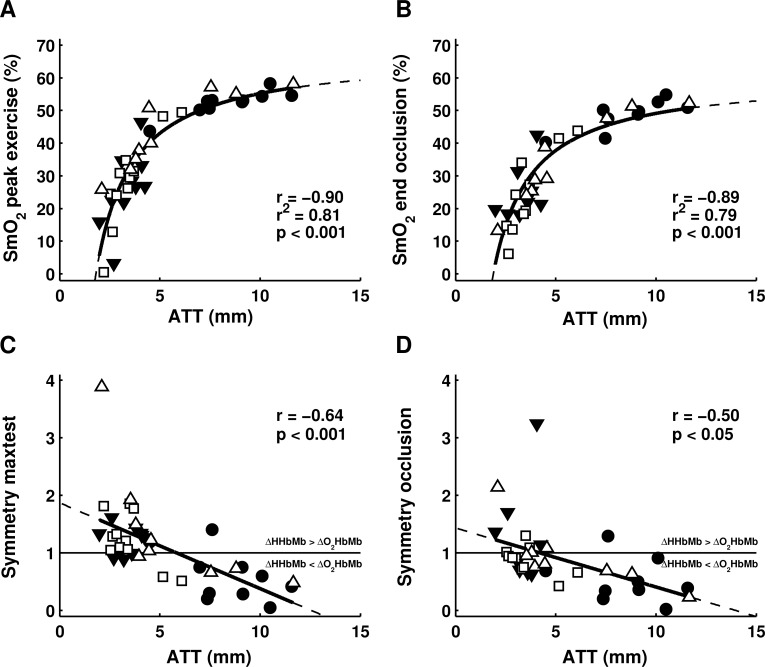
ATT affects SmO_2_ and symmetry of deoxygenated and oxygenated haemoglobin and myoglobin. Adipose tissue thickness is hyperbolically related to SmO_*2*_ at peak exercise (a) and at the end of arterial occlusion (b). Symmetry of (de)oxygenated haemoglobin (ΔHHbMb/ΔO_*2*_HbMb) during maximal exercise (c) and from rest to the end of arterial occlusion (d) are linearly related to adipose tissue thickness. Individual values are shown for male cyclists (closed triangle), female cyclists (closed circle), endurance trained males (open square), recreationally trained males (open triangle). Note that occlusion data were successfully obtained in 36 out of 40 subjects.

SmO_2_ values during exercise and occlusion were higher in groups with larger adipose tissue thickness (Table 2). Accordingly, ATT explained ~80% of SmO_2_ variance at peak exercise intensity and at the end of arterial occlusion (Fig 3). However, when SmO_2_ was normalized to correct for ATT, at VT1 (75±15.3%) and VT2 (89±8.5%) group differences disappeared. During maximal incremental exercise and occlusion, we observed asymmetrical changes in the amplitude of [HHbMb] and [O_2_HbMb] (Δ[HHbMb]/Δ[O_2_HbMb] ≠ 1). For arterial occlusion as well as for maximal incremental exercise this asymmetry was significantly related to ATT (r = -0.50, p<0.05 and r = -0.64, p<0.01 respectively; Fig 3).

## Discussion

The present findings show: 1) Δ[O_*2*_HbMb-HHbMb]-BP was not different from VT1 in male and female cyclists and recreationally trained males, but was significantly smaller than VT1 in endurance trained males, and Δ[O_*2*_HbMb-HHbMb]-BP was only moderately related to VT1 and VT2 in all groups, 2) Ventilatory thresholds showed differences across sexes and training status, whereas Δ[O_*2*_HbMb-HHbMb]-BP differed only across sexes, 3) Reproducibility was high for SmO_*2*_, Δ[O_*2*_HbMb-HHbMb]-BP and ventilatory thresholds and 4) SmO_*2*_ values were strongly affected by ATT and NIRS oxygenation present an asymmetry in [O_*2*_HbMb] and [HHbMb] that was related to ATT.

### Critical exercise threshold

The present study shows that Δ[O_*2*_HbMb-HHbMb]-BP was not significantly different from VT1 in CF, CM and RM. However, Δ[O_*2*_HbMb-HHbMb]-BP preceded VT1 in EM, which consisted of 7 male cyclists and 4 non-cyclists. Note that additional analysis revealed that in a combined group of these 7 EM cyclists and 10 CM cyclists, the Δ[O_*2*_HbMb-HHbMb]-BP was not different from VT1 (i.e. average PO value of 17 male cyclists was 224 W for Δ[O_*2*_Hb-HHb]-BP and 237 W for VT1, p>0.05). Therefore, it may be argued that in EM the Δ[O2HbMb-HHbMb]-BP was significantly smaller than VT1 due to the 4 non-cyclists (i.e. average PO of those 4 non-cyclists was 198 W for Δ[O_*2*_HbMb-HHbMb]-BP and 259 W for VT1, p<0.01). Previous studies have shown that Δ[O_*2*_HbMb-HHbMb]-BP preceded VT1 [18,19] and LT1 [18] or that Δ[O_*2*_HbMb-HHbMb]-BP was not different from LT1 [17,19]. In addition, we showed that the correlation between Δ[O_*2*_HbMb-HHbMb]-BP and ventilatory thresholds was rather moderate (r = 0.58–0.68). This deviates from previous findings in studies using similar methodology, demonstrating an excellent relationship between Δ[O_*2*_HbMb-HHbMb]-BP and VT1 or lactate threshold during stepwise incremental cycling exercise (r>0.88) [17–19]. A possible explanation for these contrasting findings is that step increments (30–50W) in the present study were larger than increments in previous studies (30W) [17–19]. Even though the regression model quality is high (r^*2*^ = 0.97), this could have led to more stepwise kinetics affecting Δ[O_*2*_HbMb-HHbMb]-BP’s goodness of fit (illustrated in Fig 1). Moreover, measurement errors related to visual inspection of ventilatory thresholds and errors related to the detection of Δ[O_*2*_HbMb-HHbMb]-BP (i.e. double linear regression model, motion artefacts, or adipose tissue (as discussed in detail below)) may have increased the unexplained variance in the relation between Δ[O_*2*_HbMb-HHbMb]-BP and VT1. Note, however, that reproducibility of Δ[O_*2*_HbMb-HHbMb]-BP and VT1 were high (ICC = 0.80–0.99). Between-subject variation, in Δ[O_*2*_HbMb-HHbMb]-BP was higher than reported in previous studies [17–19], which may be due to a more heterogeneous subject group in the present study. This heterogeneous group could have resulted in larger inter-individual variation in NIRS patterns during exercise. Note that we observed better relationships between Δ[O_*2*_HbMb-HHbMb]-BP and ventilatory thresholds in a more homogeneous group of trained cyclists (r = 0.68–0.84) as opposed to a more heterogeneous group of endurance and recreationally trained males (r = 0.48–0.50).

Physiological differences between sexes are reflected by differences in Δ[O_*2*_HbMb-HHbMb]-BP and ventilatory thresholds. The absolute exercise intensity at VT1 and Δ[O_*2*_HbMb-HHbMb]-BP (reflecting when aerobic ATP resynthesis can no longer match ATP use in the working muscles) was lower in females compared to males. The lower absolute exercise intensity in females may be explained by a lower oxygen carrying capacity and lower cardiac output [29,30,32] and possibly by smaller arteriovenous oxygen differences [29] in females. Expressed in percentage peak power output, Δ[O_*2*_HbMb-HHbMb]-BP and VT1 were found to be similar for trained male and female cyclists, indicating that relative to PO_*peak*_ the $\dot{Q}O_{2}/m\dot{V}O_{2}$ matching and consequent occurrence of increased anaerobic energy production in the muscle are similar between sexes. Previously, VT1 (in %$\dot{V}O_{2max}$) has also shown to be similar between recreationally trained males and females [10]. However, at a higher intensity exercise, the Δ[HHbMb]-BP (in %$\dot{V}O_{2max}$) has demonstrated to be lower in females compared to males, but is likely explained by less sufficient blood distribution towards active muscle in females [10].

Physiological differences between males with different training status did become apparent by differences in ventilatory thresholds, but not by differences in Δ[O_*2*_HbMb-HHbMb]-BP. In contrast, it was expected that exercise intensity (absolute and in %PO_*peak*_) at both ventilatory thresholds and Δ[O_*2*_HbMb-HHbMb]-BP would be higher in trained males, because of a higher gross efficiency in trained subjects [37] and since endurance training enhances oxidative metabolism (e.g. increases in mitochondrial density, oxidative enzymes and percentage slow-twitch myosin heavy chain types; [29,30]) and oxygen supply to the mitochondria (e.g. stroke volume, cardiac output, capillary density, haematocrit, Ca-vO_*2*_ and O_*2*_ diffusion; [29,30]). Also, subjects with a higher aerobic fitness level have been reported to demonstrate a higher percentage of slow-twitch fibres, which has been associated with more effective $\dot{Q}O_{2}/m\dot{V}O_{2}$ matching [31]. However, our results on Δ[O_*2*_HbMb-HHbMb]-BP did not confirm these expectations. Possibly, in the present study, physiological differences in training status between male groups were too small and/or measurements of Δ[O_*2*_HbMb-HHbMb]-BP were not sensitive enough to quantify differences in exercise intensity at Δ[O_*2*_HbMb-HHbMb]-BP. Although the Δ[O_*2*_HbMb-HHbMb] breakpoint is potentially a suitable exercise threshold revealing when anaerobic energy production starts to increase in the muscle, VT1 being a rather indirect measure of these changes in energy status of the muscle, discriminates better across sexes and training status in the present study.

### Reproducibility

Saturation measured by our NIRS device was obtained in trained cyclists and showed high reproducibility in rest, during exercise and at the end of arterial occlusion. These findings are similar to previously reported reproducibility values of SmO_*2*_ measurements during maximal incremental exercise (ICC = 0.81–0.95) [20], at $\dot{V}O_{2max}$ (r = 0.99) [5] and following arterial arm or leg occlusion (ICC = 0.95–0.96) [21,22]. Thus, these results imply that in trained cyclists one is able to measure oxygenation reproducibly with a continuous-wave NIRS device.

Previous results [23] showed that in sedentary subjects, breakpoints in [O_*2*_HbMb] are detected reproducibly during ramp exercise (r = 0.67–0.85, p<0.05). The present study shows that also in a group of trained cyclists the reproducibility was high for the Δ[O_*2*_HbMb-HHbMb] breakpoint (ICC = 0.80–0.88). Note that in contrast to other studies that assessed an oxygenation threshold from NIRS signals [27,38–41], the present study did not exclude any of the participating subjects from analysis. In addition, our assessment of ventilatory thresholds demonstrates excellent reproducibility (ICC = 0.96–0.99). It is well-known that ventilatory thresholds can be obtained with good reproducibility (r = 0.91–0.98) and can accurately predict endurance time trial performance [4,5]. Our reproducibility results showed that systematic measurement errors related to visual inspection of ventilatory thresholds are smaller than the measurement errors related to detection of Δ[O_*2*_HbMb-HHbMb]-BP, which may be due to effects of stepwise kinetics on the double linear regression model or effects of motion artefacts on the combined Δ[O_*2*_HbMb-HHbMb] signal. Hence, our results indicated that measurement errors related to the reproducibility of NIRS and respiratory measurements only marginally account for the unexplained variance in the relation between Δ[O_*2*_HbMb-HHbMb]-BP and VT1.

### Adipose tissue thickness

ATT is known to affect the amplitude of NIRS measurements [7,24–26,42]. Essentially, the NIRS device assumes one homogeneous medium for extending the Lambert-Beer law of light attenuation [43]. This assumption is violated by light scattering and absorption by adipose tissue [7]. Even though the VL adipose tissue thickness was below 12.5mm in all subjects of the present study, ATT comprised 11.0–64.7% of the measurement depth [24,44]. Our results confirmed that SmO_*2*_ values were substantially affected by ATT, explaining ~80% of the variance in SmO_*2*_ at peak exercise and arterial occlusion. These results indicated that NIRS saturation measurements were largely determined by ATT. Although frequently assessed by linear regressions, the relationship between NIRS signals and ATT is better described hyperbolically [22,25,26]. Clearly, the hyperbola (Fig 3) showed that SmO_*2*_ cannot be calculated for extremely low ATT values (i.e. below ~2 mm). This finding corresponds with our observation that the NIRS receiver detects no light in extremely lean subjects as the light is fully absorbed or scattered by underlying muscle tissue. Note that in extremely lean subjects the skinfold thickness predominantly consists of skin tissue. In addition to adipose tissue, cutaneous dilation in response to increased temperature will likely affect NIRS measurements as well [45]. Furthermore, lean subjects (trained males) showed SmO_*2*_ values that were roughly in line with mixed venous saturation values reported in literature at rest (~70%), VT1 (~35%) and peak exercise (15–30%) [46–48]. Thus, NIRS saturation measurements (SmO_*2*_) that are obtained from a combination of [HHbMb] and [O_*2*_HbMb] signals are clearly affected by ATT.

One may reduce effects of ATT on the amplitude of [HHbMb] and [O_*2*_HbMb] signals by normalizing oxygenation changes to the oxygenation at peak exercise [10,27], maximal voluntary contraction [26,28] or cuff occlusion [21,26]. Still, this correction does not account for the evident asymmetry in Δ[O_*2*_HbMb] and Δ[HHbMb] amplitude. Asymmetrical changes during exercise may partially be explained by blood volume changes [26,49], increased haemoglobin concentration (i.e. haemoconcentration) [50] or scattering alterations [43]. For example, continuous-wave NIRS device incorrectly assumes fixed scattering coefficients and therefore can overestimate changes in [HHbMb] (i.e. short wavelengths <750nm) during exercise [43,51]. However, the asymmetry during arterial occlusion may not be explained by changes in blood volume and haemoglobin concentration (both assumed rather constant during occlusion) or scattering coefficients (contrastingly Δ[O_*2*_HbMb] was generally larger than Δ[HHbMb]). Recent findings in canine muscle clearly showed that [O_*2*_HbMb] and [HHbMb] signals change symmetrically when no skin and adipose tissue layer is present [52]. In the present study, the asymmetry during incremental exercise as well as occlusion was significantly related to ATT. Consequently, ATT may affect the amplitude of [O_*2*_HbMb] and [HHbMb] signals to a different extent and therefore may affect kinetics when these are derived from combinations of [HHbMb] and [O_*2*_HbMb] signals (i.e. tHb: [O_*2*_HbMb+HHbMb], Hb difference: [O_*2*_HbMb-HHbMb] and SmO_*2*_). ATT may also affect determination of the Δ[O_*2*_HbMb-HHbMb]-BP if breakpoints in the individual [O_*2*_HbMb] and [HHbMb] signals do not occur at the same time. However, reproducibility of Δ[O_*2*_HbMb-HHbMb]-BP and its relationship with VT1 did not improve when the Δ[O_*2*_HbMb-HHbMb] signal was corrected for the observed asymmetry in [O_*2*_HbMb] and [HHbMb] amplitude (results not presented). Hence, ATT scattering and absorbance likely affect [O_*2*_HbMb] and [HHbMb] amplitude differently, but it remains to be established exactly how this occurs and whether this may confound the detection of Δ[O_*2*_HbMb-HHbMb]-BP.

## Conclusions

During maximal incremental step exercise, the Δ[O_2_HbMb-HHbMb] breakpoint is reproducible and coincides with VT1, but is only moderately related to the ventilatory thresholds. Although the Δ[O_2_HbMb-HHbMb] breakpoint is potentially a suitable exercise threshold revealing when anaerobic energy production starts to increase in the muscle, the VT1 (being a more indirect measure of these changes in muscle energy status) shows higher reproducibility and discriminates better across sexes and training status. Continuous-wave NIRS measurements are reproducible, but adipose tissue thickness strongly affects the amplitude of [O_2_HbMb] and [HHbMb] signals to a different extent and thereby may also affect the kinetics of combined [O_2_HbMb] and [HHbMb] signals. The large inter-individual variability observed at the Δ[O_2_HbMb-HHbMb] breakpoint and its underlying nature is yet poorly understood, although some of this variability may be explained by differences in adipose tissue thickness. Correcting the Δ[O_2_HbMb-HHbMb] breakpoint for ATT however is difficult, since it remains to be established how ATT affects the absorbance by O_2_HbMb and HHbMb at the measured wavelengths. Therefore, the first ventilatory threshold is favoured over the Δ[O_2_HbMb-HHbMb] breakpoint in context of determining an exercise threshold that reflects the metabolic demands of active muscle in maximal stepwise incremental exercise.

## Supporting Information

S1 File Supporting excel-file with data of the present study. (XLSX)

## Abbreviations

Δ[O_2_HbMb-HHbMb]-BP: oxygenation breakpoint, obtained from subtracting concentration changes in deoxygenated haemoglobin and myoglobin from the oxygenated haemoglobin and myoglobin
ATT: adipose tissue thickness
Ca-vO_2_: arterio-venous oxygen differences
CF: trained female cyclists
CM: trained male cyclists
CO_2_: carbon dioxide
EM: endurance trained males
HHbMb: deoxygenated haemoglobin and myoglobin
$m\dot{V}O_{2}$: oxygen utilization, muscle oxygen uptake
NIRS: near-infrared spectroscopy
O_2_: oxygen
O_2_HbMb: oxygenated haemoglobin and myoglobin
PO: power output
PO_2_: oxygen pressure
$\dot{Q}O_{2}$: oxygen supply
RM: recreationally trained males
SmO_2_: muscle saturation
$\dot{V}O_{2}$: oxygen uptake
$\dot{V}O_{2max}$: maximal oxygen uptake
VT1: first ventilatory threshold
VT2: second ventilatory threshold; respiratory compensation point
